# Dietary Modulation of Migraine: Metabolic, Neuroinflammatory and Microbiota-Mediated Mechanisms

**DOI:** 10.3390/jcm15041476

**Published:** 2026-02-13

**Authors:** Domenico Santangelo, Concetta Lobianco, Rosalia Eugenia Burrafato, Federico Tosto, Giuseppe Magro, Angelo Pascarella

**Affiliations:** 1 Unit of Neurology, Sant’Elia Hospital, 93100 Caltanissetta, Italy; domenicosnt92@gmail.com (D.S.); clobianco@virgilio.it (C.L.); 2 Istituto Tolman, 90139 Palermo, Italy; rosaliaburrafato@live.it; 3 Department of Neuroscience, “Giovanni Paolo II” Hospital, 88046 Lamezia Terme, Italy; tostofederico@hotmail.it (F.T.); giuseppemagro.neuro@gmail.com (G.M.); 4 Department of Medical and Surgical Sciences, Magna Graecia University, 88100 Catanzaro, Italy; 5 Regional Epilepsy Centre, Great Metropolitan “Bianchi-Melacrino-Morelli Hospital”, 89124 Reggio Calabria, Italy

**Keywords:** DASH diet, dietary interventions, gut microbiota, gut–brain axis, headache, inflammation, ketogenic diet, mitochondrial dysfunction, omega-3 fatty acids, oxidative stress

## Abstract

Migraine is a common neurological condition characterized by recurrent headache attacks, frequently associated with prodromal, aura, and postdrome phases. Increasing evidence suggests that metabolic and mitochondrial dysfunction play a central role in migraine pathophysiology, contributing to cortical hyperexcitability and increased oxidative stress. Additionally, the gut microbiota has emerged as an important modulator of migraine susceptibility via the gut–brain axis, influencing inflammation, neurotransmitter production, and neuronal excitability. Specific dietary interventions, including ketogenic diets, low-carbohydrate diets, DASH, omega-3 supplementation, and elimination diets, may modulate these metabolic and inflammatory pathways, as well as the microbiota composition, ultimately reducing the frequency and severity of migraine attacks. This review provides an overview of current evidence on the interplay between metabolism, microbiota, and diet in migraine pathophysiology and management. Overall, the available data support a biologically plausible role for diet as an adjunctive strategy in migraine prevention; however, the current evidence remains highly heterogeneous and is often limited by small sample sizes in sample size, a lack of protocol standardization, suboptimal adherence assessment, and insufficient long-term follow-up. Future studies should focus on adequately powered trials with standardized outcome measures, objective biomarkers and precision medicine approaches.

## 1. Introduction

Migraine is a chronic, disabling neurovascular disorder characterized by recurrent episodes of moderate-to-severe headache, typically unilateral and pulsating in quality, and frequently associated with nausea, vomiting, photophobia, and phonophobia. In approximately one-third of patients, migraine attacks are preceded or accompanied by transient focal neurological symptoms, known as aura, which most frequently manifests as visual disturbances but may also involve sensory, language, or motor symptoms [1]. Migraine affects approximately 15% of the global population and represents one of the leading causes of years lived with disability across all age groups [2]. Despite its high prevalence and clinical impact, migraine remains a complex and not yet fully elucidated disorder. Early theories primarily attributed its pathogenesis to primary vascular dysregulation. According to the classical vascular hypothesis, intracranial vasoconstriction was responsible for the aura, followed by reactive extracranial vasodilation responsible for headache onset [3]. However, this perspective has progressively evolved toward a more complex neurobiological model. Central to modern migraine pathophysiology theory is neurogenic inflammation [4], specifically the activation of the trigeminovascular system, which innervates intracranial blood vessels and the dura mater. The activation of this system leads to the release of vasoactive neuropeptides, including Calcitonin Gene-Related Peptide (CGRP), substance P, and neurokinin A, leading to vasodilation, plasma protein extravasation, and neurogenic inflammation. These processes result in peripheral and central sensitization of nociceptive pathways, thereby amplifying pain perception and contributing to migraine chronicization [5].

A growing body of evidence indicates that patients with migraine show impaired energy metabolism and reduced metabolic flexibility [6]. This metabolic vulnerability is further exacerbated by cortical spreading depression (CSD), a slowly propagating wave of profound neuronal and glial depolarization that represents the electrophysiological substrate of migraine aura. CSD imposes an exceptionally high and massive metabolic demand on the brain, potentially exceeding oxygen and glucose availability and triggering a cascade of oxidative stress and inflammatory signaling, transient tissue hypoxia, ionic imbalance and glutamate release [7]. Within this framework, migraine attacks can be conceptualized as the clinical manifestation of a failure in adaptive homeostatic mechanisms occurring when cumulative metabolic, oxidative and inflammatory stress exceeds an individual threshold. Lifestyle factors such as fasting, dehydration, sleep disturbances, psychological stress, alcohol intake and hormonal fluctuations can further reduce this threshold by increasing energetic demand or oxidative burden [6]. Among modifiable lifestyle factors, diet occupies a uniquely central role, as it directly influences cerebral energy metabolism, mitochondrial efficiency, redox balance, inflammatory tone, neurotransmitter synthesis, and ion channel stability [8]. In parallel, the gut–brain axis has emerged as a critical mediator connecting dietary factors to migraine susceptibility [9]. Alterations in gut microbiota composition (i.e., dysbiosis) and impaired intestinal barrier integrity may facilitate systemic inflammation and alter microbial metabolite production, further lowering the migraine threshold [10]. Collectively, these findings provide a strong biological rationale for investigating dietary modulation as a therapeutic strategy in migraine. Current preventive pharmacological therapies, including, more recently, monoclonal antibodies targeting CGRP or its receptor, are effective only in a proportion of patients and are often limited by adverse effects, contraindications, or long-term tolerability issues. Dietary interventions represent a promising complementary option, supported by growing clinical evidence that specific regimens can reduce migraine attack frequency and severity [11].

This review aims to synthesize current evidence on dietary modulation in migraine, integrating metabolic, neuroinflammatory and microbiota-mediated mechanisms, and to provide a comprehensive mechanistic framework supporting the use of diet as an adjunctive or preventive approach in migraine management.

## 2. Materials and Methods

For this narrative review, a comprehensive literature search was performed using PubMed/MEDLINE and Google Scholar databases to identify relevant articles published up to November 2025. The search strategy combined Medical Subject Headings (MeSH) terms and free-text keywords related to migraine, diet, metabolism, and gut microbiota. In details, the primary search terms included “migraine” and “diet”, “metabolism”, “microbiota”, “gut–brain axis”, “dietary intervention”, “DASH”, “ketogenic diet”, “nutrition”, “gut microbiota”, “mitochondrial dysfunction”, “oxidative stress”, and “chronic migraine”. These terms were used alone and in various combinations to maximize sensitivity. Randomized controlled trials, non-randomized interventional studies, observational studies, systematic reviews, meta-analyses, and mechanistic experimental studies relevant to migraine pathophysiology and dietary modulation were included. Priority was given to randomized controlled trials, meta-analyses, and well-designed observational or mechanistic studies when available. Areas supported primarily by preclinical or small clinical studies were discussed with appropriate caution. When conflicting results were identified, findings were interpreted considering differences in study design, population characteristics, dietary protocols, outcome measures, and duration of follow-up. Duplicates were removed during the screening process, and studies were excluded when they were clearly outside the topic. No date restrictions were applied. Only articles published in English were included. The reference lists of selected articles and key reviews were also screened to identify additional pertinent studies. The final reference list included all available original studies and selected reviews based on importance, originality, quality, and relevance to the purpose of this review.

## 3. Results

### 3.1. Migraine and Metabolic Vulnerability

**Figure 1:**
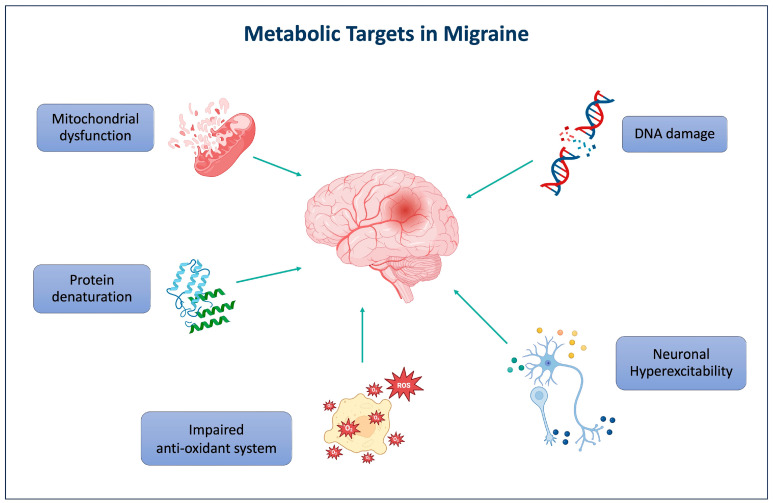
Metabolic and inflammatory mechanisms in migraine pathogenesis. The figure illustrates major metabolic and molecular mechanisms implicated in migraine pathogenesis, including mitochondrial dysfunction, oxidative stress, protein and DNA damage and neuronal hyperexcitability, all of which contribute to disease initiation and progression.

A recent growing body of evidence supports the notion that migraine is associated with a state of chronic cerebral metabolic vulnerability, detectable even during interictal periods and characterized by abnormalities in mitochondrial function and cerebral energy metabolism (Figure 1). Advanced neuroimaging techniques, particularly phosphorus-31 magnetic resonance spectroscopy (^31^P-MRS), have provided compelling evidence of altered cerebral energy metabolism in migraine. Both migraine with and without aura are characterized by reduced phosphocreatine and ATP levels and impaired oxidative phosphorylation, indicating unstable cerebral energetics [12,13]. Recent multimodal neuroimaging studies using proton magnetic resonance spectroscopy (^1^H-MRS) and resting-state functional MRI have revealed dynamic changes in cerebral metabolism across the migraine cycle, demonstrating phase-specific alterations in neuronal integrity, membrane turnover, neuronal mitochondrial dysfunction and altered neurotransmitter balance [14,15]. PET imaging studies also suggest dysfunction within thalamocortical circuits, particularly in chronic migraine, potentially contributing to central sensitization and disease persistence [16].

Inadequate energy supply markedly increases susceptibility to CSD, positioning the mitochondrial–oxidative stress–CSD axis as a central pathway in migraine pathogenesis [17]. Neurons, especially within the cerebral cortex, operate with minimal energetic reserve and rely heavily on efficient mitochondrial ATP production to maintain ion gradients, synaptic transmission, and neurotransmitter recycling. When mitochondrial efficiency is compromised, neurons require disproportionate metabolic input to sustain baseline activity, reducing the threshold for pathological events such as CSD. Restoration of ionic gradients following CSD is extremely energy-intensive, requiring rapid activation of ATP-dependent pumps and metabolic pathways [18]. Experimental studies have demonstrated that CSD induces transient tissue hypoxia, mitochondrial dysfunction, and excessive production of reactive oxygen species (ROS), further challenging cellular energy reserves [19,20]. Nitric oxide (NO) release during CSD further exacerbates mitochondrial dysfunction, as high NO concentrations inhibit multiple components of the mitochondrial respiratory chain, impair ATP synthesis, and promote oxidative stress. Damage to mitochondrial respiration in any brain region may contribute to a global energy deficit, supporting the view that migraine represents a response to cerebral energy deficiency exceeding antioxidant capacity [6,18]. CSD itself is a potent inducer of ROS formation in the cerebral cortex, meninges, and trigeminal ganglia. ROS contribute to both central and peripheral sensitization by modulating protein kinase activity, altering glutamatergic transmission, regulating ion channels such as Transient Receptor Potential Vanilloid 1 (TRPV1) and Transient Receptor Potential Ankyrin 1 (TRPA1), and promoting neurogenic inflammation [21]. ROS can directly activate nociceptive receptors and facilitate the release of CGRP from peptidergic afferents, reinforcing trigeminovascular activation. Experimental evidence suggests that inhibition of ROS production and modulation of TRPA1 channels may attenuate stress-induced migraine by reducing CGRP-mediated signaling [21]. Astrocytes may play a critical role. The dysregulation of astrocyte–neuron lactate shuttling may compromise energy availability during sustained neural activation and further lower the threshold for CSD. From a broader perspective, migraine can be conceptualized as a disorder characterized by a reduced capacity of the nervous system to adapt to metabolic and environmental stressors [22].

This framework also explains the frequent coexistence of migraine with metabolic comorbidities such as insulin resistance, obesity, and sleep disorders, all of which are associated with impaired mitochondrial function and systemic inflammation [23]. Mitochondrial dysfunction impairs glucose-stimulated insulin secretion and contributes to hyperinsulinemia, which, in turn, exacerbates oxidative stress and neuronal excitability [24]. Case–control studies have shown that migraine patients exhibit exaggerated insulin responses to glucose loads and higher levels of insulin resistance, which correlate with increased attack frequency and severity [24,25]. Hyperinsulinemia further promotes oxidative stress and mitochondrial dysfunction, thereby facilitating CSD and migraine onset [26]. Experimental evidence indicates that neurotransmitters and vasoactive mediators released during CSD, including NO and CGRP, interact bidirectionally with mitochondrial function. Elevated NO and CGRP levels impair mitochondrial respiration, reduce ATP production, and increase ROS generation, while mitochondrial dysfunction itself enhances NO and CGRP expression, creating a self-reinforcing pathological loop [23].

### 3.2. Neuroinflammation as a Consequence of Metabolic Stress

Neuroinflammation represents a central mechanistic link between metabolic dysfunction and pain generation in migraine. Inflammation and immune activation play a significant role in migraine pathophysiology. Elevated levels of inflammatory mediators, including IL-6, TNF-α, and CGRP, have been implicated in transmission, vascular dysregulation, and central sensitization during migraine attacks. Signaling molecules released by resident and peripheral immune cells, including T lymphocytes and macrophages, contribute to maintaining a pro-inflammatory environment that exacerbates nociceptive processing [27].

Inflammatory signaling emerges as a downstream response to metabolic and energetic stress within susceptible neural networks. This perspective reconciles metabolic vulnerability, CSD, and trigeminovascular activation into a unified pathophysiological framework. Activation of the trigeminovascular system is a defining feature of migraine attacks. Primary afferent neurons originating in the trigeminal ganglion innervate intracranial blood vessels and the dura mater, and their activation leads to the release of vasoactive neuropeptides, most prominently CGRP, but also substance P and neurokinin A [28]. CGRP induces potent vasodilation, promotes plasma protein extravasation, and facilitates peripheral and central sensitization of nociceptive pathways, thereby amplifying pain perception. However, accumulating evidence suggests that CGRP release may represent, at least in part, an adaptive response to metabolic and oxidative stress. CGRP is widely expressed in sensory neurons and has been shown to exert cytoprotective, antioxidant, and vasoregulatory effects in multiple tissues. Experimental studies demonstrate that CGRP attenuates ROS production, enhances endothelial function, and supports cellular survival under ischemic or metabolic stress conditions [29]. This dual role of CGRP aligns with the concept of migraine as a disorder of impaired homeostatic resilience. When metabolic stress exceeds compensatory capacity, protective signaling pathways are activated to restore tissue integrity and energy balance. Vasodilation mediated by CGRP may serve to enhance cerebral perfusion and substrate delivery, while its antioxidant effects may limit oxidative damage. Supporting this view, experimental data show that ROS promote CGRP production in rodent models of CSD induced by potassium chloride stimulation and can restore cortical susceptibility to CSD following CGRP inhibition [21].

Metabolic stress is a potent trigger of neuroinflammatory signaling within the central nervous system (CNS). Impaired mitochondrial function leads to ATP depletion, disruption of ion gradients, and accumulation of extracellular potassium and glutamate. These changes promote the release of ATP and other danger-associated molecular patterns, which in turn activate microglia and astrocytes [30]. CSD represents a critical event linking metabolic stress to neuroinflammation. Experimental models have shown that CSD triggers the release of inflammatory mediators, activates microglia, and induces long-lasting changes in meningeal nociceptor sensitivity [20,30]. The metabolic demands imposed by CSD, combined with transient hypoperfusion and oxidative stress, further amplify inflammatory signaling. Repeated episodes of CSD may therefore contribute to cumulative neuroinflammatory burden and facilitate migraine chronification. CSD also upregulates the expression of cyclooxygenase-2 (COX-2), TNF-α, interleukin-1β (IL-1β), and matrix metalloproteinases (MMPs). Activation of MMPs transiently compromises blood–brain barrier (BBB) integrity, altering the extracellular milieu of the cerebral cortex and increasing potassium, hydrogen ions, nitric oxide, and epinephrine concentrations. These changes sensitize or directly excite ipsilateral trigeminovascular afferents, further facilitating headache generation [31].

Oxidative stress plays a central role in this process. Mitochondrial dysfunction results in excessive production of reactive oxygen species, which can directly modify ion channels, alter neurotransmitter release, and activate redox-sensitive transcription factors such as NF-κB. These pathways promote the expression of pro-inflammatory genes and perpetuate a state of low-grade neuroinflammation [6].

The interaction between metabolic stress, neuroinflammation, and CGRP signaling may also help explain interindividual variability in migraine expression and treatment response. Patients with greater metabolic impairment or oxidative burden may exhibit enhanced CGRP release and heightened inflammatory signaling, rendering them more responsive to CGRP-targeted therapies [32]. Dietary factors exert a profound influence on neuroinflammatory tone. Diets rich in refined carbohydrates, trans fats, and ultra-processed foods promote systemic inflammation and oxidative stress, whereas diets rich in antioxidants, omega-3 fatty acids, and polyphenols exert anti-inflammatory effects. Ketogenic diets, in particular, reduce neuroinflammation through inhibition of the NLRP3 inflammasome and modulation of microglial activation [32]. The reinterpretation of CGRP signaling as a context-dependent adaptive response also has implications for long-term treatment strategies. While CGRP monoclonal antibodies are effective in reducing migraine frequency, their impact on protective vascular and metabolic functions remains an area of active investigation. Understanding how dietary interventions may reduce the need for compensatory CGRP release by improving metabolic resilience could inform more balanced, integrative treatment approaches.

### 3.3. Microbiota and Migraine

In recent years, the gut–brain axis has emerged as a critical interface linking dietary factors, metabolic regulation, immune signaling, and CNS function [33]. The gut microbiota, the complex community of microorganisms inhabiting the gastrointestinal tract, is hypothesized to play a central role in modulating processes directly relevant to migraine susceptibility [34]. Moreover, migraine patients exhibit a significantly higher prevalence of gastrointestinal comorbidities compared with the general population [10]. Both migraine and functional gastrointestinal disorders are characterized by visceral hypersensitivity, altered autonomic regulation, low-grade inflammation, and stress-related symptom exacerbation [35]. Diet is the primary determinant of microbiota composition, making nutritional interventions a powerful modulator of gut–brain signaling. Dysbiosis, defined as an imbalance in microbial diversity, composition, or function, has been increasingly reported in migraine populations, although findings vary depending on study design and analytical methods [34]. Metagenomic studies have identified differences in bacterial taxa between migraine patients and healthy controls, including alterations in genera involved in inflammatory regulation, short-chain fatty acid (SCFA) production, and nitric oxide metabolism. One proposed mechanism linking dysbiosis to migraine involves immune activation. The intestinal epithelium acts as a selective barrier, preventing translocation of microbial products into the circulation. Disruption of this barrier (often referred to as increased intestinal permeability or “leaky gut”) allows bacterial components such as lipopolysaccharides (LPS) to enter the bloodstream, triggering low-grade systemic inflammation. Circulating inflammatory mediators from a “leaky gut” may amplify the pre-existing neuroinflammatory tone, further lowering the trigeminovascular activation threshold [36]. Much of the mechanistic insight into these stems from experimental models. In a study by Souza et al. (2004), germ-free mice subjected to intestinal ischemia–reperfusion injury exhibited reduced inflammatory responses and higher anti-inflammatory cytokine IL-10 levels [37]. However, the direct translatability of these animal findings to human migraine pathophysiology requires further validation. Stress represents an additional factor linking gut microbiota and migraine. Stress-induced dysbiosis may amplify inflammatory and metabolic disturbances, through activation of the hypothalamic–pituitary–adrenal (HPA) axis. By modulating cortisol secretion, HPA axis activation can alter intestinal permeability and immune signaling, thereby facilitating microbial translocation and inflammatory responses [38]. Importantly, bidirectional communication within the gut–brain axis allows neural circuits themselves to regulate HPA axis activity, underscoring the dynamic interaction between central stress responses and peripheral immune-microbial mechanisms [35]. In the gut, immune cells release inflammatory mediators, including IL-1, IL-6, TNF-α, and C-reactive protein, which are associated with increased attack severity [39].

Another important microbiota-mediated pathway involves NO signaling. Several oral and gut bacteria possess nitrate-reducing capabilities, converting dietary nitrates into nitrites and ultimately NO. Alterations in the abundance or activity of these bacteria may influence systemic NO availability and vascular reactivity [40]. Metagenomic analyses provide indirect evidence of this link showing an overrepresentation of nitrate-, nitrite-, and nitric oxide-reducing bacteria in migraine patients, such as species within the genera Rothia, Neisseria, and certain Proteobacteria, suggesting alterations in NO-reducing bacteria may exacerbate the vascular reactivity and trigeminovascular sensitization [34,40].

Short-chain fatty acids (SCFAs) represent a link between the gut microbiota and the CNS. Produced via bacterial fermentation of dietary fiber, SCFAs, including acetate, propionate, and butyrate, are essential for intestinal eubiosis and epithelial integrity [41]. Beyond local effects, SCFAs exert neuroprotective roles by modulating the systemic and central inflammatory pathways identified as key migraine drivers [42]. Reduced SCFA production, as observed in dysbiotic states, may impair microglial homeostasis and promote a pro-inflammatory CNS environment. Migraine has been associated with decreased abundance of SCFA-producing bacteria, particularly butyrate-producing taxa such as Ruminococcaceae and Faecalibacterium lineages, suggesting that impaired microbial metabolite availability may contribute to neuroinflammatory priming and a lower migraine threshold [34]. Experimental studies in animals suggest that SCFA deficiency may enhance neuroinflammatory responses and stress-induced behavioral and sensory alterations, supporting a plausible but to be confirmed role for SCFA-mediated mechanisms in modulating migraine susceptibility [34].

The gut microbiota also influences tryptophan metabolism and serotonin signaling, pathways highly relevant to migraine. Approximately 90% of the body’s serotonin is produced in the gastrointestinal tract, and gut bacteria regulate tryptophan availability and its metabolic fate. Altered serotonin signaling has long been implicated in migraine pathophysiology, affecting both vascular tone and nociceptive processing. Dysbiosis-induced alterations in tryptophan metabolism may therefore have a role in serotonergic dysregulation in migraine [34,43].

Clinical evidence further supports the potential role of microbiota alterations in migraine, including in pediatric populations. Data from the American Gut Project indicate that children and adolescents with migraine exhibit reduced microbial diversity and increased abundance of inflammation-associated taxa, such as Eggerthella, Sutterella, and Eubacterium, while non-migraine controls show higher levels of Firmicutes, particularly Christensenellaceae and Ruminococcaceae [44]. Eggerthella has been linked to bacteremia, gastrointestinal disease, and neuropsychiatric disorders, suggesting a pathogenic profile, whereas Ruminococcaceae may exert protective effects via butyrate production, starch fermentation, promotion of beneficial microbial networks, and negative correlations with inflammatory markers [34,44]. Sutterella is associated with mucosal immune activation and altered IgA responses, indicating a role in immune–microbiota dysregulation rather than direct pathogenicity. A recent systematic review reported consistent associations between migraine and altered microbial diversity, inflammatory-related taxa, and microbial metabolic pathways, though studies showed substantial heterogeneity and a lack of longitudinal or mechanistic human data [34]. At the taxonomic level, migraine patients repeatedly show depletion of beneficial commensals, particularly butyrate-producing genera within Firmicutes (e.g., Ruminococcaceae and Lachnospiraceae), which support intestinal barrier integrity and exert anti-inflammatory effects. Conversely, pro-inflammatory taxa, including Eggerthella, Sutterella, and certain Enterobacteriaceae, are enriched, reinforcing the systemic inflammatory tone [34].

Dietary interventions offer a means of modulating these microbiota-mediated mechanisms. Western-style diets high in refined carbohydrates, saturated fats, and ultra-processed foods favor dysbiosis and inflammatory signaling [45,46]. Diets rich in dietary fiber, polyphenols, and fermented foods promote microbial diversity and SCFA production. Similarly, ketogenic and low-carbohydrate diets induce marked shifts in gut microbiota composition, some of which may contribute to their anti-inflammatory and neuroprotective effects [47]. Probiotic and prebiotic interventions have been explored as potential migraine therapies, although evidence remains preliminary. Small, randomized trials suggest that probiotic supplementation may reduce migraine frequency and severity, potentially by modulating inflammatory markers and improving gut barrier function [48]. However, improvements in migraine symptoms are often linked to changes in inflammatory markers rather than consistent microbial shifts, suggesting that anti-inflammatory effects may precede or outweigh microbiota restructuring. Overall, current evidence for microbiota-targeted strategies is less robust than for other metabolic interventions. Most clinical studies in this area are limited by small sample sizes, short duration, and non-standardized endpoints, making it difficult to draw definitive conclusions for clinical practice.

### 3.4. Dietary Interventions in Migraine

Dietary interventions represent the most direct and clinically applicable strategy for modulating the metabolic, neuroinflammatory, and microbiota-mediated mechanisms implicated in migraine pathophysiology. Unlike pharmacological therapies that typically target downstream nociceptive pathways, dietary strategies exert pleiotropic effects on upstream biological processes, including cerebral energy metabolism, oxidative stress, inflammatory signaling, and gut–brain axis communication [8]. Pro-inflammatory dietary patterns can contribute to systemic inflammation, for instance via metabolic endotoxemia, where gut-derived lipopolysaccharides from an altered microbiota increase circulating cytokines such as TNF-α and IL-6 [9,45,46]. These mediators can compromise BBB integrity by disrupting tight junction proteins, promoting neuroinflammation and lowering the threshold for trigeminovascular activation. Although certain foods have been reported as migraine triggers, the core pathophysiological is driven by endogenous trigeminovascular activation, independent of dietary intake. Changes in circulating neuropeptides and inflammatory mediators may thus reflect broader metabolic effects rather than diet per se. For instance, reduction in substances such as CGRP could be an indirect consequence of improved metabolic homeostatic, such as glycemic profile stabilization and reduced systemic low-grade inflammation and may also be influenced by concurrent lifestyle factors including weight loss, caloric restriction, hydration and physical activity.

In the following sections, we report the main evidence from the literature on dietary interventions for migraine management. The clinical evidence supporting these dietary approaches varies significantly. For example, while high-level evidence from randomized controlled trials is available for ketogenic diets, strategies such as probiotic supplementation or specific elimination diets currently rely on a more limited evidence base. Although several mechanistic pathways have been proposed, most dietary intervention trials did not systematically assess inflammatory or oxidative stress biomarkers; therefore, these mechanisms should be interpreted as biologically plausible assumptions rather than directly demonstrated effects. Table 1 provides an overview of the randomized controlled trials (i.e., placebo-controlled, active-controlled, sham-controlled and cross-over study) available to date for each dietary approach.

#### 3.4.1. Ketogenic Diet and Ketone-Based Metabolic Therapy

The ketogenic diet (KD) is characterized by a marked reduction in carbohydrate intake, moderate protein consumption, and high fat intake, resulting in a metabolic shift from glucose-based energy production toward fatty acid oxidation and hepatic ketogenesis. Ketone bodies (primarily β-hydroxybutyrate and acetoacetate) serve as efficient alternative energy substrates for the brain and exert multiple neurobiological effects relevant to migraine. From a metabolic perspective, ketone bodies enhance mitochondrial efficiency by increasing ATP yield per unit of oxygen consumed and stabilizing neuronal energy supply. β-hydroxybutyrate has been shown to reduce mitochondrial reactive oxygen species production and improve redox balance, thereby directly addressing the oxidative stress associated with migraine [87]. In addition, ketone bodies modulate neuronal excitability by influencing ATP-sensitive potassium channels and glutamatergic neurotransmission, mechanisms that may raise the threshold for CSD. Ketogenic diets also exert anti-inflammatory effects. β-hydroxybutyrate inhibits the NLRP3 inflammasome, reducing the production of pro-inflammatory cytokines such as interleukin-1β [32]. Furthermore, ketone metabolism influences gene expression by inhibiting histone deacetylases, thereby promoting antioxidant and cytoprotective pathways [43,88].

Interest in KD for migraine dates back to early research in the 20th century, reporting reduction on frequency and intensity of migraines attacks, but modern evidence has renewed its relevance. A pivotal observation by Di Lorenzo et al. (2013) demonstrated migraine remission during ketogenic phases and relapse during non-ketogenic periods in twin patients [89]. Subsequent cohort and randomized studies confirmed rapid and significant reductions in migraine frequency, intensity, and disability with ketogenic regimens [49,52,90], with consistent real-world validation in refractory populations [91,92]. The rapid onset of benefit suggests a direct neurobiological mechanism beyond weight loss. Nevertheless, not all studies have reported consistent results. Haslam et al. (2021) found no significant differences between KD and an anti-headache dietary regimen, with a high rate of side effects and limited tolerability [50]. Adherence and tolerability remain critical limitations, particularly in adolescents [50]. A double-blind RCT study in 2022 investigating exogenous ketone supplementation failed to replicate ketogenic efficacy, suggesting that ketosis alone may be insufficient and that associated metabolic changes contribute to clinical benefit [51]. Neurophysiological studies support the central cortical mechanism of KD. Evidence indicates that KD normalizes cortical habituation deficits, suggesting that cortical networks may represent the primary site of ketogenic modulation [49]. As regards long-term efficacy, data are particularly encouraging. A sustained reduction in migraine frequency, duration, intensity, and medication use after 6 and 12 months of a modified Atkins ketogenic diet, together with improvements in metabolic and inflammatory markers and without major safety concerns has been demonstrated [93]. However, long-term adherence to strict ketogenic regimens can be challenging, and potential adverse effects necessitate careful patient selection and monitoring.

#### 3.4.2. Low-Carbohydrate and Low Glycemic Index Diets

Less restrictive dietary approaches, such as low-carbohydrate and low glycemic index (LGI) diets, aim to stabilize blood glucose levels without inducing full nutritional ketosis. These diets reduce postprandial glucose and insulin fluctuations, which are known to influence neuronal excitability and susceptibility to CSD. Hypoglycemia and rapid glucose declines are frequently reported migraine triggers, supporting the relevance of glycemic control. LGI diets promote more stable glucose availability and reduce reliance on rapid glycolytic flux, thereby lowering metabolic stress. A clinical trial has shown that LGI diets is associated with significant reductions in migraine frequency, duration, and pain intensity, with benefits sustained over long-term follow-up [53]. These improvements occur independently of weight loss, reinforcing the concept that metabolic stabilization rather than caloric restriction per se is the key therapeutic factor.

Low-carbohydrate diets may also exert indirect anti-inflammatory effects by reducing insulin-mediated activation of pro-inflammatory pathways and improving mitochondrial substrate utilization. Compared with ketogenic diets, LGI approaches are generally more sustainable and may represent a first-line dietary intervention for many migraine patients [94,95]. Hyperinsulinemia and insulin resistance are coupled to stress-kinase signaling, which can amplify cytokine output and further impair insulin receptor substrate function; reducing dietary carbohydrate load may therefore dampen this self-reinforcing inflammation–insulin loop [94,96]. In migraine cohorts, individualized low-glycemic programs supported by continuous glucose monitoring have reported reductions in migraine/headache days and disability measures [95,96].

#### 3.4.3. Antioxidant-Rich and Anti-Inflammatory Dietary Patterns

The Dietary Approaches to Stop Hypertension (DASH) diet exemplifies this approach. Originally developed for cardiovascular health, the DASH diet is rich in magnesium, potassium, folate, vitamin C, and polyphenols, all of which have been implicated in migraine modulation. Randomized controlled trials have demonstrated that adherence to the DASH diet significantly reduces migraine frequency, duration, and disability compared with control diets, independent of weight loss [54].

Omega-3 polyunsaturated fatty acids are another key dietary component relevant to migraine. Omega-3 fatty acids modulate inflammatory lipid mediators by shifting eicosanoid production toward less pro-inflammatory and pro-resolving pathways. Controlled trials have shown that increased omega-3 intake, particularly when combined with reduced omega-6 consumption, leads to modest but significant reductions in migraine frequency and severity [55]. These effects are accompanied by reductions in headache-related inflammatory biomarkers.

Mediterranean-style dietary patterns, which incorporate many of these anti-inflammatory components, have also been associated with lower migraine prevalence and reduced headache burden in observational studies, although high-quality interventional data remain limited [97]. An 8-week Mediterranean–ketogenic pilot study in chronic migraine reported early decreases in frequency and intensity alongside improvements in insulin resistance markers, providing preliminary proof-of-concept for Mediterranean-compatible, metabolically oriented interventions [98]. In adults with migraine, higher Mediterranean diet adherence scores have been associated with lower attack frequency and shorter duration, as well as reduced headache impact/disability indices [99].

#### 3.4.4. Elimination Diets

Elimination diets based on immune-mediated food sensitivities have been proposed as therapeutic strategies in migraine, particularly in patients reporting food-related triggers or gastrointestinal symptoms. Several studies have explored the use of elimination diets guided by food-specific immunoglobulin G (IgG) antibodies. Randomized trials have demonstrated that removal of foods associated with elevated IgG antibodies can reduce migraine frequency and severity in selected patients [56,57,58,59]. While the immunological significance of IgG-mediated food sensitivity remains controversial, elimination diets may reduce exposure to dietary components that promote inflammation, gut permeability, or metabolic stress. More recently, a sham-controlled randomized trial reported that excluding IgG-positive foods over 12 weeks improved migraine symptom measures and was accompanied by reductions in inflammatory mediators, suggesting that dietary immune signals may intersect with systemic inflammation and trigeminovascular sensitization [60]. A randomized crossover intervention combining a low-fat, plant-based diet with an elimination phase targeting common trigger foods reduced headache frequency, duration, intensity, and medication use, supporting the broader concept that simplifying dietary exposures may benefit some individuals [61]. However, excessive dietary restriction risks nutritional deficiencies and reduced adherence, underscoring the importance of individualized, supervised approaches.

#### 3.4.5. Micronutrient Supplementation and Personalized Nutrition

In recent years, nutraceuticals have emerged as preventative treatments for migraine in both adults and pediatric patients [100]. Several micronutrients, including magnesium, riboflavin (vitamin B2), coenzyme Q10, and folate, play critical roles in mitochondrial energy metabolism, endothelial function, and neuronal excitability [6].

Among nutraceuticals, magnesium has been the most extensively studied. Magnesium modulates NMDA receptor activity, calcium channel conductance, and nitric oxide synthesis, thereby influencing cortical excitability and trigeminovascular activation [101]. Randomized controlled trials have demonstrated that oral magnesium supplementation significantly reduces migraine frequency and intensity compared with placebo, particularly in patients with low baseline magnesium levels [62,63,65,66,67,68]. Otherwise, two RCT have failed to show a decrease in the number of migraine attacks and migraine days [64,69], although reduction in Migraine Disability Assessment Score Questionnaire (MIDAS) and Headache Impact Test-6 (HIT-6) was found [73]. Several recent systematic reviews have provided evidence for effectiveness of magnesium for prophylactic treatment of migraine, suggesting that a dose of 600 mg daily represent a safe and cost-efficient strategy in clinical practice [101,102,103].

Riboflavin supports mitochondrial ATP production and reduces oxidative stress, providing a biological rationale for its use in migraine prevention. High-dose riboflavin (400 mg/day) significantly reduced migraine frequency and headache days in a landmark randomized controlled trial [70], with consistent confirmation in subsequent studies, systematic reviews and meta-analyses [73,83,85,104]. In pediatric populations, results are less consistent. Low-dose riboflavin (10–40 mg/day) showed modest benefit in a retrospective cohort [105], whereas placebo-controlled trials using 50–200 mg/day failed to demonstrate consistent efficacy [71,72], although reductions in tension-type headache features were observed [72]. Conversely, Talebian et al. (2018) reported significant reductions in migraine frequency and duration with high-dose riboflavin in children [74]. Overall, riboflavin efficacy appears to depend on dosage, age, and headache phenotype. Therefore, while riboflavin is recommended for adult migraine prevention, its role in pediatric migraine remains insufficiently supported.

Several evidence supports the role of coenzyme Q10 (CoQ10) as a safe and effective adjunct in migraine prevention. Randomized and controlled studies consistently demonstrate reductions in migraine frequency, severity, and duration following CoQ10 supplementation, alongside improvements in inflammatory and oxidative stress markers [75,76,78]. Pediatric data further indicate comparable efficacy to amitriptyline with superior tolerability [77]. Combination formulations including CoQ10, magnesium, riboflavin, and feverfew show additional benefits on pain intensity and disability scores, suggesting potential synergistic mitochondrial effects, although the isolated contribution of CoQ10 cannot be fully determined [85,106]. Improvements in mitochondrial metabolic markers, such as plasma lactate, further support the central role of mitochondrial dysfunction in migraine pathophysiology [86]. Overall, CoQ10 appears to represent a well-tolerated, biologically plausible, and clinically relevant option within personalized migraine prevention strategies [107].

More recently, vitamin D has emerged as an additional micronutrient of interest. Several randomized controlled trials and case–control studies have reported that vitamin D supplementation reduces migraine frequency, headache days, and inflammatory markers, particularly in patients with baseline deficiency [79,80,81,82]. These effects may be mediated through modulation of neuroinflammation, CGRP expression, and immune regulation [108].

Folate and B-vitamin supplementation is particularly relevant in migraine with aura. Methylenetetrahydrofolate reductase (MTHFR) polymorphisms are associated with hyperhomocysteinemia, endothelial dysfunction, and increased migraine susceptibility [84]. Elevated homocysteine may promote CSD and vascular instability. Clinical trials and meta-analyses demonstrate that combined supplementation with folic acid, vitamin B6, and vitamin B12 reduces homocysteine levels and improves migraine outcomes in genetically susceptible patients [84,109].

## 4. Future Directions

**Figure 2:**
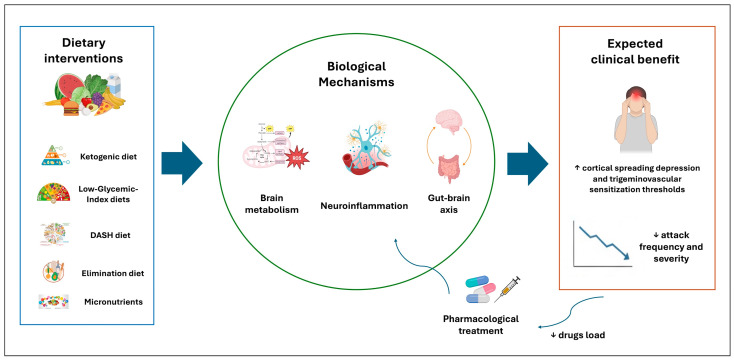
Conceptual framework of dietary modulation in migraine. The figure summarizes the proposed mechanisms through which dietary interventions modulate migraine susceptibility. Dietary strategies influence metabolic resilience, neuroinflammatory pathways, and gut–brain axis signaling, thereby increasing the threshold for cortical spreading depression and reducing trigeminovascular sensitization. These convergent effects may translate into a reduced migraine burden and cold represent a helpful strategy complementing pharmacological therapies (Created with BioRender.com).

The present review integrates a rapidly expanding body of evidence supporting the concept that migraine is a complex neurological disorder rooted in impaired metabolic resilience, neuroinflammatory activation, and altered gut–brain axis signaling. The evidence reviewed indicates that dietary interventions influence all three core dimensions of migraine pathophysiology: metabolism, neuroinflammation, and microbiota-mediated signaling (Figure 2) [110,111]. Ketogenic and low-carbohydrate diets primarily target cerebral energy metabolism and oxidative stress, whereas antioxidant-rich and anti-inflammatory diets modulate immune signaling and redox balance. Elimination diets and microbiota-targeted strategies further address gut–brain axis dysfunction. Rather than competing approaches, these dietary strategies may be conceptualized as points along a continuum of metabolic intervention. Crucially, dietary strategies should be considered adjunctive interventions within a comprehensive migraine care model, alongside established pharmacological treatments (such as CGRP-targeting therapies), rather than as stand-alone alternatives.

However, important gaps remain. Many dietary intervention studies are limited by small sample sizes, short follow-up periods, variability in protocols, and challenges with adherence [34,111]. While clinical support is strongest for ketogenic or low-glycemic strategies and selected nutraceuticals, where randomized data exist, evidence for microbiota-targeted interventions remains preliminary and heterogeneous, with a current level of evidence is substantially lower than for established metabolic therapies. Disentangling the effects of metabolic changes, weight loss, and placebo responses is often difficult, which constrains the generalizability and interpretability of findings [110]. Future large-scale, standardized randomized trials should include standardized dietary protocols, objective biomarkers of metabolic and inflammatory status and robust patient-reported outcome measures. Advances in metabolomics, microbiomics, and neuroimaging may offer unprecedented opportunities to identify metabolic phenotypes, microbiota signatures, and genetic variants that predict responsiveness to dietary interventions. Importantly, clinical benefits do not always correspond to measurable surrogate markers, such as microbial shifts or systemic cytokine levels. As observed in other areas of pain medicine, therapeutic effects may extend beyond anatomically or radiologically apparent changes, suggesting that benefits arise from complex, systemic modulations rather than a single mechanistic pathway. Finally, long-term safety and sustainability of chronic dietary modulation, particularly ketogenic or low-carbohydrate strategies, require careful evaluation, particularly regarding the impact on cardiovascular risk and micronutrient status.

In our view, dietary modulation should be considered a valuable adjunct intervention within standard migraine care and not primary therapeutic strategy, applied through a stepwise approach guided by feasibility and risk–benefit balance. Low-glycemic and anti-inflammatory dietary patterns can be considered the default starting point for many patients, given their favorable tolerability profile and broader applicability, whereas ketogenic regimens should be reserved for selected refractory cases and implemented only under close clinical monitoring, to address significant adherence, tolerability and long-term sustainability [49,50,51,52,54,55,90,91,92,93,94,95,96,97,98,99]. Broad elimination approaches should not be recommended as routine clinical practice; when considered, they should be highly individualized (e.g., in patients with relevant gastrointestinal comorbidities) and supervised to avoid unnecessary dietary restriction and nutritional imbalance [56,57,58,59,60,61]. Importantly, we emphasize that any dietary intervention should be framed as a time-limited therapeutic trial with predefined clinical endpoints and standardized headache outcomes. The interpretation of benefit should be cautious, acknowledging the significant confounding effects of weight loss, and expectancy responses, which frequently can confound perceived benefit [34,110,111].

## 5. Conclusions

Recent evidence indicate that migraine should be conceptualized as a multisystem disorder in which attacks arise when cumulative metabolic, inflammatory, and gut–brain stress exceeds the adaptive capacity of the brain. Dietary interventions represent a biologically grounded, patient-centered strategy that can complement pharmacological therapies, potentially reducing attack frequency, severity, and reliance on medications. Dietary strategies should be framed not as replacements for evidence-based pharmacological treatments but as complementary tools that may enhance overall efficacy, reduce medication burden, and empower patients through lifestyle-based self-management. Personalized approaches, informed by metabolic phenotype, microbiota composition, and genetic variability, may optimize clinical efficacy and long-term sustainability. Overall, dietary modulation represents a promising integrative strategy for migraine prevention and management.

## Figures and Tables

**Table 1:** Review of the literature on randomized controlled trial investigating dietary intervention in migraine patients.

Author, Date	Type of Study	Patients	Interventions (Dose)	Study Duration	Concomitant Drug Prophylaxis	Primary Outcomes	Results
**Ketogenic diet**							
Di Lorenzo et al., 2019 [49]	Randomized, controlled, double-blind, cross-over trial	35 adults	VLCKD vs. nonketogenic VLCD	4 weeks	No	Monthly migraine days; migraine attacks; acute medication use	- Migraine days ↓ by −3.73 with VLCKD vs. VLCnKD (*p* < 0.001); - ≥50% responder rate 74.3% vs. 8.6%; migraine attacks ↓ by −3.02 (*p* < 0.001); - no difference in acute medication use
Haslam et al., 2021 [50]	Randomized controlled crossover trial	16 adults	Ketogenic diet vs. healthy anti-headache diet	12 weeks	n.a.	Headache days; headache severity; headache duration	- No significant between-diet differences in headache days/severity; - Headache duration ↓ in ketogenic arm
Putananickal et al., 2022 [51]	Randomized, placebo-controlled, double-blind crossover	38 adults (16 verum, 22 placebo)	7.54 g β-hydroxybutyrate vs. placebo	Each treatment period: 12 weeks	Yes	Migraine days; migraine intensity	- No significant differences between the two groups
Caprio et al., 2023 [52]	Randomized controlled trial	57 adults (30 VLCKD, 29 controls)	VLCKD vs. HBD	24 weeks	No	Monthly migraine days; quality of life	- Migraine days ↓ significantly with VLCKD at weeks 8, 12, and 24 - QoL ↑ with VLCKD at weeks 8 and 12
**Low-Glycemic-Index Diets**							
Evcili et al., 2018 [53]	Randomized controlled trial	304 adults (147 verum, 147 controls)	Low glycemic index vs. controls	3 months	Yes (in controls)	Migraine severity (VAS)	- No differences between groups at 1 month. - At 3 months, significantly higher VAS ↓ in low-GI diet group.
**DASH**							
Arab et al., 2022 [54]	Parallel group randomized controlled trial	102 adult females (51 DASH, 51 controls)	DASH diet (7-day menu cycle; ~15–20% protein, 25–30% fat, 55–60% carbs) vs. usual dietary advice (control)	12 weeks	Yes	Migraine frequency, severity and duration	- Greater reduction in migraine frequency (−3.00 vs. −1.40; *p* = 0.025) and severity (−1.76 vs. −0.59; *p* < 0.001) with DASH; - Trend toward reduced duration (*p* = 0.053).
Ramsden et al., 2013 [55]	Randomized controlled trial	182 participants (61 H3 diet, 61 H3-L6 diet, 60 control diet)	Three different diets groups: (1) H3 diet (EPA+DHA 1.5 g/day, linoleic acid at 7% of energy); (2) H3-L6 diet (EPA+DHA 1.5 g/day, linoleic acid ≤ 1.8% of energy); (3) control diet (EPA+DHA < 150 mg/day, linoleic acid at 7%)	12 weeks	Yes	Headache days; headache hours; HIT-6	- Both H3-L6 and H3 diets ↓ total headache hours/day, moderate–severe headache hours/day, and headache days/month vs. control. - H3-L6 reduced headache days/month more than H3. - No significant differences in HIT-6 score
**Elimination Diets**							
Alpay et al., 2010 [56]	Double-blind, randomized, controlled, cross-over clinical trial	30 adult patients	IgG elimination diet vs. provocation diet	6 weeks	Yes	Headache days; migraine attacks frequency	- Significant reduction in headache days and migraine attacks during the elimination diet compared with provocation diet (both *p* < 0.001).
Mitchell et al., 2011 [57]	Sham-controlled randomized clinical trial	167 adults (84 true elimination diet, 83 sham diet)	IgG elimination diet vs. placebo (sham diet)	12 weeks	Yes	Migraine frequency	- Slightly, non-significant, ↓ migraine-like headaches in the elimination diet group compared to placebo group (*p* = 0.18).
Aydinlar et al., 2013 [58]	Double-blind randomized controlled cross-over trial	21 adults with migraine and IBS	IgG-based individualized elimination diet vs. a provocation diet (control)	n.a.	Yes	Attack frequency; severity; QoL	- Significant ↓ in attack frequency, maximum and mean duration, maximum severity, and acute medication use significantly with elimination diet vs. baseline (all *p* ≤ 0.01). - Elimination diet ↓ pain–bloating severity and frequency and ↑ QoL (*p* < 0.05) compared with provocation diet
Xie et al., 2019 [59]	Randomized controlled trial	60 patients with migraine and IBS	IgG elimination diet vs. probiotics vs. diet combined with probiotics	14 weeks	Yes	Migraine days; MIDAS	- Migraine days ↓ significantly with elimination diet at 14 weeks and with elimination diet + probiotics at 7 and 14 weeks; - No significant change with probiotics alone. - MIDAS score ↓ at 7 weeks only in elimination + probiotics group. At 14 weeks, MIDAS ↓ significantly in all groups vs. baseline.
Zhao et al., 2025 [60]	Sham-controlled randomized trial	98 adults (52 true IgG elimination diet, 46 sham diet)	IgG elimination diet vs. placebo (sham diet)	12 weeks	No	VAS; MIDAS; inflammatory markers	- Higher ↓ in VAS and MIDAS scores and ↓ IgG, IL-6, TNF-α and CGRP in true elimination diet group vs. sham. - Between-group difference in migraine days
Bunner et al., 2014 [61]	Randomized, controlled, crossover trial	42 adults	Low-fat vegan diet (4 weeks) followed by elimination diet vs. placebo supplement	16-week periods with 4-week washout	Yes	VAS; headache intensi); headache frequency; patient’s Global Impression of Change	- Worst headache pain ↓ 2.1 cm diet vs. 0.7 cm supplement (*p* = 0.03) - Average headache intensity ↓ 1.0 diet vs. 0.5 supplement (*p* = 0.20) - Headache frequency ↓ 0.3 diet vs. 0.4 supplement (*p* = 0.61) - Patient’s Global Impression of Change showed greater improvement with diet (*p* < 0.001)
**Micronutrient**							
**Magnesium**							
Facchinetti et al. 1991 [62]	Double-blind, placebo-controlled study	20 females with menstrual migraine *	Mg pyrrolidone carboxylic acid (oral, 360 mg/day) vs. placebo	2 months	n.a.	Headache days; MDQ score; PTI	- Headache days and MDQ scores ↓ with Mg. - PTI ↓ in both placebo and Mg, with lower values in Mg group
Peikert et al., 1996 [63]	Multi-center, placebo-controlled and double-blind randomized study	81 adults (43 intervention, 38 placebo)	TriMg dicitrate (oral, 600 mg/day) vs. placebo	12 weeks	No	Attack frequency; attack duration; headache intensity; number of drugs	- Attack frequency significantly more ↓ in Mg (41.6%) than placebo (15.8%) group (*p* < 0.05). - No difference in attack duration, intensity, and drug use per attack
Pfaffenrath et al., 1996 [64]	Randomized, double-blind, placebo-controlled study	69 (35 intervention, 34 placebo)	Mg aspartate hydrochloride (oral, providing 486 mg elemental Mg/day) vs. placebo	12 weeks	No	≥50% responders; migraine days	- No significant difference in ≥50% responders (28.6% in Mg group, 29.4% in placebo group), migraine days and frequency. - Adverse events ↑ with Mg (45.7% vs. 23.5%, mainly mild gastrointestinal symptoms)
Wang et al., 2003 [65]	Randomized, double-blind, placebo-controlled trial	86 children (42 intervention, 44 placebo)	Mg oxide (oral, 9 mg/kg per day) vs. placebo	16 weeks	No	Headache frequency; severity	- Headache frequency ↓ over time with Mg (*p* = 0.0037) but not with placebo (*p* = 0.086) - No significant between-group difference (*p* = 0.88). - Headache severity significantly ↓ in Mg vs. placebo group (*p* = 0.0029).
Köseoglu et al., 2008 [66]	Double blind, randomized, placebo-controlled study	40 adults (30 intervention, 10 placebo); 20 healthy controls for VEP analysis	Mg citrate (oral, providing 600 mg elemental Mg/day) vs. placebo	3 months	No	Attack frequency; severity; VEP parameters; cerebral blood flow	- Attack frequency, severity, and VEP P1 amplitude ↓ with Mg vs. baseline (*p* < 0.001). - Post/pre ratios ↓ significantly in Mg vs. placebo group. - Cortical blood flow ↑ in frontal, temporal, and insular regions with Mg; no change with placebo.
Tarighat Esfanjani et al., 2012 [67]	Randomized single-blinded, controlled trial	133 adults (33 Mg, 35 L-carnitine, 30 Mg+L-carnitine, 35 controls)	Mg oxide (oral, 500 mg/day) vs. L-carnitine (500 mg/day) vs. Mg+L-carnitine vs. controls	12 weeks	Yes	Migraine attacks; severity	- Migraine attacks/month, migraine days/month, and headache severity ↓ significantly in all groups (*p* < 0.05). - Independent significant effect of Mg in repeated measures and nested model.
Karimi et al., 2021 [68]	Randomized, controlled, double-blind crossover trial	63 adults (31 Mg, 32 VPA)	Mg oxide (oral, 500 mg/day) vs. VPA (oral, 400 mg b.i.d.)	8 weeks each trial period, 4-week washout	No	Migraine frequency; migraine days; attack duration	- No difference in attack frequency, migraine days and attack duration between Mg and VPA group
Khani et al., 2021 [69]	Randomized single-center double-blind parallel-group controlled clinical trial	222 adults (82 VPA; 70 Mg+VPA; 70 Mg)	VPA (200 mg b.i.d.) vs. VPA (200 mg b.i.d) + Mg oxide (250 mg b.i.d) vs. Mg oxide (250 mg b.i.d)	12 weeks	No	Headache frequency; severity; duration; MIDAS; HIT-6	- Headache frequency, attack severity and duration, monthly analgesic use ↓ significantly vs. baseline in all groups (*p* < 0.001). - Similar headache frequency between VPA and VPA+Mg; - Severity, duration, and analgesic use ↓ more with VPA+Mg vs. VPA (*p* < 0.05). Poorer efficacy in Mg vs. both VPA and VPA+Mg (*p* < 0.001). - MIDAS and HIT-6 ↓ in all groups, with greater improvement in VPA and VPA+Mg vs. Mg (*p* < 0.001).
**Riboflavine**							
Schoenen et al., 1998 [70]	Randomized controlled trial	55 adults (28 verum, 27 placebo)	Riboflavin (oral, 400 mg) vs. placebo	3 months	n.a.	Attack frequency; headache days	- Significant ↓ in attack frequency and headache days in riboflavin vs. placebo. - Higher ≥50% responder rate in riboflavin (59%) than placebo (15%) group (*p* = 0.002).
MacLennan et al., 2008 [71]	Double-blind, randomized, placebo-controlled trial	48 children (27 verum, 21 placebo)	Riboflavin (oral, 200 mg/day) vs. placebo	3 months	No	Responder rate; severity, duration, nausea/vomiting days, or analgesic use	- No significant differences between groups for ≥50% responder rate (44.4% with riboflavin vs. 66.7% with placebo), migraine severity, duration, nausea/vomiting days, or analgesic use.
Bruijn et al., 2010 [72]	Randomized, placebo-controlled, double-blind, cross-over trial	42 children	Riboflavin (oral, 50 mg/day) vs. placebo	10 months	No	Migraine frequency	- No significant difference in migraine attack frequency between riboflavin and placebo (*p* = 0.44). - Tension-type headache frequency ↓ significantly with riboflavin vs. placebo (*p* = 0.04).
Rahimdel et al. 2015 [73]	Randomized controlled trial	98 adolescents (50 verum, 48 VPA)	Riboflavin (oral, 400 mg/day) vs. VPA (oral, 500 mg/day)	3 months	No	Headache frequency; duration; severity; adverse events	- Headache frequency, monthly median duration and severity ↓ in both riboflavin and VPA groups with no significant between-group differences. - Adverse effects ↓ significantly with riboflavin vs. VPA (*p* = 0.005)
Talebian et al., 2018 [74]	Randomized, double-blind, placebo-controlled trial	90 children (30 placebo, 30 low-dose riboflavin and 30 high-dose riboflavin)	Low-dose riboflavin (oral, 100 mg(day) vs. high-dose riboflavin (200 mg day) vs. placebo	12 weeks	No	Migraine frequency; duration; intensity	- Significant ↓ in migraine frequency and mean duration in high-dose riboflavin vs. placebo group (both *p* = 0.000). - No significant differences between low-dose riboflavin and placebo. - No between-groups difference for migraine intensity.
**Coenzyme Q10**							
Sándor et al., 2005 [75]	Double-blind, randomized, placebo-controlled trial.	42 adults (21 verum, 21 placebo)	CoQ10 (oral, 3 × 100 mg/day) vs. placebo	4 months	No	Attack frequency; headache days; nausea days	- Significant ↓ in attack frequency, headache days, and nausea days with CoQ10 vs. placebo at month 3. - Significant higher ≥50% responder rate in CoQ10 (47.6%) vs. placebo (14.4%) group
Slater et al., 2011 [76]	Randomized, double-blinded, placebo-controlled, crossover, add-on study	150 pediatric patients	CoQ10 (oral, 100 mg/day) vs. placebo	224 days	Yes	Migraine frequency; severity	- Migraine frequency, severity, and duration ↓ in both CoQ10 and placebo groups (all *p* < 0.05). - No differences between the CoQ10 and placebo groups at day 224.
Yaghini et al., 2022 [77]	Randomized, active-controlled, clinical trial	72 children (36 CoQ10, 36 amitriptyline)	CoQ10 (oral, was 30 mg for <30 kg patients and 60 mg for ≥ 30 kg patients) vs. amitriptyline (oral, 1–2 mg/kg)	3 months	No	Migraine frequency, duration, severity; QoL (Pediatric MIDAS)	- Both treatments improved migraine outcomes; no significant difference after 3 months - Amitriptyline acted faster, but more side effects - QoL improved similarly in both groups
Dahri et al. 2023 [78]	Randomized double-blind placebo-controlled clinical trial	46 females (23 verum, 22 placebo)	CoQ10 (oral, 400 mg/day) vs. placebo	12 weeks	Yes	Migraine frequency; severity; duration; inflammatory markers	- Significant ↓ in migraine frequency, severity, and duration with CoQ10 vs. placebo. - CGRP and TNF-α ↓ significantly with CoQ10; no changes in IL-6 and IL-10
**Vitamin D**							
Buettner et al., 2015 [79]	Randomized, double-blind, placebo-controlled trial	57 adults (28 verum, 29 placebo)	Vitamin D3 (oral, 1000 UI/day) + simvastatin (oral, 20 mg/die) vs. placebo	24 weeks	Yes	Migraine days; ≥50% responder rate	- Migraine days ↓ significantly more with simvastatin + vitamin D vs. placebo at weeks 1–12 and 13–24 (both *p* < 0.001). - ≥50% responders: 25% at 12 weeks and 29% at 24 weeks vs. 3% with placebo (*p* = 0.03). - Comparable adverse events.
Gazerani et al., 2019 [80]	Randomized, double-blinded, placebo-controlled parallel trial	48 adults (24 verum, 24 placebo)	Vitamin D3 (oral, 100 μg/day) vs. placebo	24 weeks	Yes	Headache days; migraine frequency, severity	- Significant ↓ in headache days and migraine frequency with vitamin D vs. placebo at 24 weeks (*p* < 0.001). - No significant between-group differences for migraine severity.
Ghorbani et al., 2020 [81]	Randomized placebo-controlled, double-blind study	80 adults (40 verum, 40 placebo)	Vitamin D3 (oral, 2000 IU) vs. placebo	12 weeks	Yes	Frequency, attack duration, severity, analgesic use	- Significant ↓ in headache days/month, attack duration, headache severity, analgesic use with vitamin D3 vs. placebo (all *p* < 0.05). - iNOS serum levels ↓ significantly with vitamin D3; - No significant difference in IL-6, IL-10 and Cox-2
Tirani et al., 2024 [82]	Randomized, triple-blinded, placebo-controlled trial	72 adults (36 vit. D3 + probiotics, 36 placebo)	Vitamin D (50,000 IU/2 weeks) + probiotic (4.5 × 10^11^ CFU/day) vs. placebo	12 weeks	Yes	Migraine frequency, severity, duration, hs-CRP, DASS, HIT-6 scores	- Significant ↓ in migraine frequency and severity with probiotic + vitamin D vs. placebo. No significant between-group differences for headache duration, hs-CRP, DASS, or HIT-6 scores.
**Combinations**							
Maizels et al., 2004 [83]	Randomized double-blind placebo-controlled trial	49 adults (24 verum, 25 placebo)	Treatment with riboflavin (400 mg) + Mg (300 mg) + feverfew (100 mg) vs. placebo (riboflavin 25 mg)	3 months	Yes	Migraine frequency, migraine days, migraine index, triptan use	- No significant differences between groups for migraine frequency (42% vs. 44%, *p* = 0.87), migraine days (33% vs. 40%, *p* = 0.63), migraine index, or triptan use
Lea et al., 2009 [84]	Randomized, double-blind, placebo-controlled trial	52 adults (37 verum, 15 placebo)	Vitamin supplementation with folic acid (oral, 2 mg/day) + vitamin B6 (oral, 25 mg/day) + vitamin B12 (oral, 400 μg/day) vs. placebo	6 months	Yes	Migraine frequency, migraine severity, homocysteine levels	- Homocysteine: ↓ 39% in verum (*p* = 0.001) vs. 20% in placebo (*p* > 0.05) - Migraine severity: ↓ 61% → 30% in verum (*p* = 0.01) vs. no significant change in placebo (*p* = 0.3) - Headache frequency: median 4 → 1 in verum (*p* = 0.04) vs. no change in placeb0 (*p* > 0.05) - Headache severity: median 6 → 4.5 in verum (*p* = 0.002) vs. no change in placebo (*p* > 0.05)
Gaul et al., 2015 [85]	Randomized, placebo-controlled, double-blind, multicenter trial	112 adults (55 verum, 57 placebo)	Treatment (riboflavin 400 mg + Mg 600 mg + CoQ10 150 mg + multivitamin/trace elements) vs. placebo	3 months	No	Migraine days; HIT-6	- Migraine days/month ↓ in both groups, with no significant difference (*p* = 0.23). - Significantly higher ↓ in pain intensity (*p* = 0.03), HIT-6 score (*p* = 0.01), and patient-reported efficacy (*p* = 0.01) in treatment vs. placebo group.
Hajihashemi et al., 2019 [86]	Randomized, double-blind, placebo-controlled, study	56 adults	CoQ10 (oral, 30 mg/day) + L-carnitine (oral, 500 mg/day) vs. placebo	8 weeks	No	Headache severity, duration, frequency, serum lactate levels	- Significant ↓ in headache severity, duration and frequency in the intervention group (*p* ≤ 0.001) - Significant serum lactate ↓ in the intervention group (*p* = 0.002)

References are ordered by publication date. * Number of patients for each group not reported. CoQ10: coenzime Q10; DASH: Dietary Approaches to Stop Hypertension; DASS: Depression, Anxiety, and Stress Scales; DHA: docosahexaenoic acid; HBD: hypocaloric balanced diet; EPA: eicosapentaenoic acid; HIT-6: Headache Impact Test-6; IBS: irritable bowel syndrome; MIDAS: Migraine Disability Assessment Score Questionnaire; MDQ: Menstrual Distress Questionnaire scores; n.a.: not available; PTI: Pain Total Index; VAS: visual analog scale; VLCKD: very low-calorie ketogenic; VPA: valproic acid.

## Data Availability

No new data were created or analyzed in this study. Data sharing is not applicable to this article.

## Abbreviations

The following abbreviations are used in this manuscript:

^1^H-MRS	proton magnetic resonance spectroscopy
^31^P-MRS	phosphorus-31 magnetic resonance spectroscopy
ADP	adenosine diphosphate
ATP	adenosine triphosphate
CGRP	Calcitonin Gene-Related Peptide
CoQ10	coenzyme Q10
CSD	cortical spreading depression
DASH	Dietary Approaches to Stop Hypertension
HIT-6	Headache Impact Test-6
IgG	immunoglobulin G
IL-6	interleukin-6
KD	ketogenic diet
LGI	low glycemic index
LPS	lipopolysaccharides
MeSH	Medical Subject Headings
MIDAS	Migraine Disability Assessment Score Questionnaire
MTHFR	Methylenetetrahydrofolate reductase
NAA	N-acetylaspartate
NO	nitric oxide
PCr	phosphocreatine
ROS	reactive oxygen species
SCFA	short-chain fatty acids
tCr	total creatine
TRPV1	Transient Receptor Potential Vanilloid 1
TRPA1	Transient Receptor Potential Ankyrin 1
TNF-α	tumor necrosis factor-α
